# Secreted autotransporter toxin produced by probiotic *Escherichia coli* Nissle 1917 enhances neurodegeneration in *Caenorhabditis elegans*

**DOI:** 10.17912/micropub.biology.001366

**Published:** 2025-01-30

**Authors:** Graham Redweik, Ding Xue

**Affiliations:** 1 Department of Molecular, Cellular and Developmental Biology, University of Colorado Boulder, Boulder, United States

## Abstract

First isolated during World War I, *Escherichia coli* Nissle 1917 (ECN) is an intensively studied bacterium that produces several factors that can inhibit pathogenic bacteria to promote gut health. These findings have led to its commercialization as a probiotic bacterium widely available for human consumption. Notably, the genome of ECN is highly analogous to many extraintestinal pathogenic *E. coli* (ExPEC) strains that do cause diseases in humans. Both ECN and ExPEC carry the *sat* gene which encodes a cytotoxic serine autotransporter toxin (Sat). Given that the role of ECN Sat in human disease has been poorly studied, we sought to implement the nematode *C. elegans* as a model for analyzing how ECN Sat may impact human neurodegenerative disease. Using multiple *C. elegans* disease models, we find that ECN Sat induces significantly higher neurodegeneration in several *C. elegans* models we tested. Although preliminary, our early findings suggest that careful studies are paramount to assess the impact of ECN to humans susceptible of neurodegenerative disease to determine the long-term safety of ECN as a commercial probiotic.

**Figure 1. Escherichia coli Nissle 1917 Sat enhances severity in multiple C. elegans neurodegenerative disease models:**
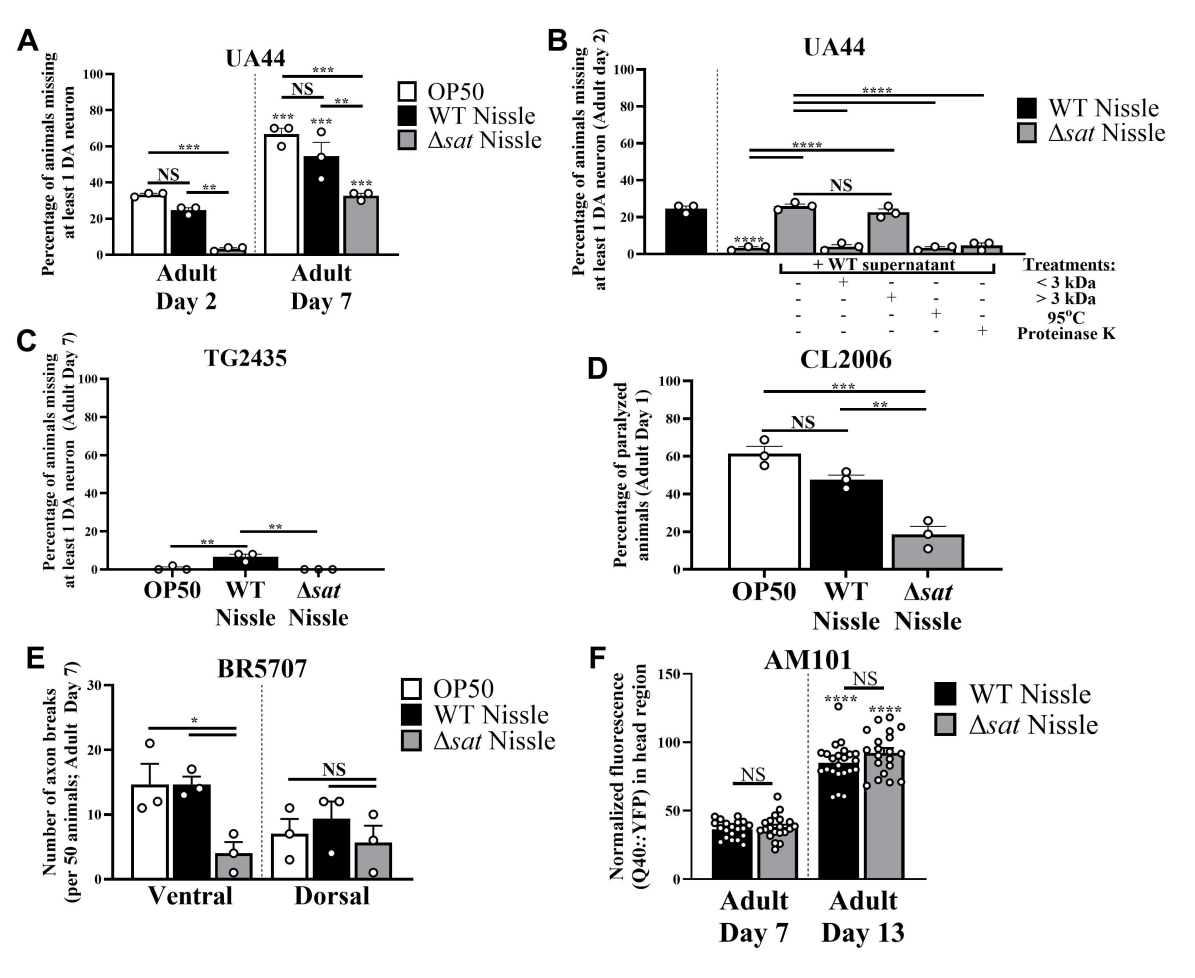
We used multiple *C. elegans* neurodegenerative disease models, including Parkinson's disease (Figures 1A-C: UA44 , TG2435 ), Alzheimer's disease (Figure 1D: CL2006 ), tauopathy (Figure 1E: BR5707 ), and Huntington's disease (Figure 1F: AM101 ), to examine the impact of ECN Sat. Consistently, the presence of ECN Sat exacerbates the disease phenotypes in most models tested. For Figures 1A-C, neurodegeneration was quantified by determining the loss of at least one dopaminergic neuron in the head region via fluorescent microscopy. For Figure 1D, paralysis was determined for each animal via lack of movement with and without gentle-prodding with a worm pick. For Figure 1E, axonal breaks in dorsal and ventral cords were evaluated via fluorescent microscopy. For Figure 1F, polyglutamine (Q40) levels in head neurons were quantified for each animal via fluorescent microscopy and ImageJ analysis. * *P* < 0.05; ** *P* < 0.01; *** *P* < 0.001; **** *P* < 0.0001. One-way ANOVAs followed by Tukey's multiple comparisons tests were performed to determine statistical differences between bacterial diets, age groups, and supernatant treatments. Asterisks over bars indicate significance of difference between identical treatment groups at different developmental stages, whereas asterisks above the lines indicate significance of difference between different treatment groups at the same stage or same side of the axon cord. For Figures 1A-E, results are presented as the mean of three trials (50 animals per trial), whereas in Figure F, data points indicate each animal evaluated (15 animals per biological replicate), respectively.

## Description

*E. coli* Nissle 1917 (ECN; serotype O6:K5:H1), originally isolated from the feces of a World War I German soldier uniquely resistant to a shigellosis outbreak, has been found to compete against pathogenic intestinal pathogens like *Salmonella* and *Shigella* via iron sequestration, antimicrobial peptide production, and other colonization resistance factors (reviewed in Effendi and Ng, 2023). These properties of ECN promote gut health by protecting against intestinal pathogens, making it one of the earliest probiotic bacteria to ever be used to improve human health. Sold as the commercial product Mutaflor® (Jacobi and Malfertheiner, 2011) , ECN has demonstrated experimental and clinical success in promoting intestinal health (Pradhan and Weiss, 2020; reviewed in Scaldaferri et al., 2016) . Furthermore, genetically-modified ECN have shown efficacy against cancer and obesity (reviewed in Effendi and Ng, 2023), suggesting this bacterium may have wide utility to combat several diseases. However, one important consideration for the therapeutic implementation of ECN is that this *E. coli* strain is genetically-similar to extraintestinal pathogenic *E. coli* (ExPEC) (Grozdanov et al. *,* 2004). Notably, ECN is generally considered to be safe for consumption given that this bacterium lacks the genes encoding hemolysin and P-fimbriae (Grozdanov et al. *,* 2004), crucial for extraintestinal survival in the bloodstream (reviewed in Sora et al., 2021). Despite lacking these ExPEC virulence factors, ECN does carry the ExPEC gene *sat* , which encodes the secreted autotransporter toxin (Sat) (Grozdanov et al. *,* 2004). Sat, a 107-kDa secreted serine protease, plays an important role in *E. coli* pathogenesis via immune evasion (Freire et al., 2022) and has cytotoxic activity (Vieira et al., 2020) . Similarly, ECN Sat alone disrupts paracellular permeability in intestinal cells (Toloza et al., 2015) , although other secreted factors from ECN appear to antagonize Sat activity (Toloza et al., 2015) . Despite the explicit claim that, based on these findings, ECN Sat is not a virulence factor, ECN Sat did cause significant toxicity to undifferentiated epithelial cells (Toloza et al., 2015) , supporting the notion that ECN Sat could be harmful to human cells. Since most studies of ECN have been mainly focused on its intestinal effects, there has been virtually no study investigating its impact on neurological diseases like Parkinson's disease (PD) and Alzheimer's disease, many of which are late onset and may take years to show symptoms. Similarly, despite the cytotoxic effects of Sat, no study has investigated its impact on neurological diseases. Notably, 16S rRNA levels of *Enterobacteriaceae,* including *Escherichia* , are higher in the gut microbiomes of Parkinson's patients compared to healthy controls (Li et al *.* , 2023), suggesting that intestinal *E. coli,* which display high homology to ECN (Conway and Cohen, 2015) and similarly carry *sat* (Toloza et al., 2015) , may directly contribute to PD pathogenesis. Thus, we investigated whether ECN Sat contributes to neurodegeneration, using *Caenorhabditis elegans* neurodegenerative disease models.

Using the PD model, UA44 , which co-expresses GFP and human α-synuclein (αS), an aggregation prone protein associated with PD, in dopaminergic (DA) neurons under the control of the *dat-1* gene promoter (Kautu et al., 2013) , we find that wild-type (WT) ECN induces similar levels of neurodegeneration compared to *E. coli* OP50 (serotype B and uracil auxotroph), the standard diet for *C. elegans* ( Figure 1A ) that, notably, does not possess *sat* (BioProject PRJNA41499 ). Approximately 25-30% of adult day 2 animals and 55-65% of adult day 7 animals lost at least one of the six dopaminergic neurons labeled by GFP in the head region. However, animals fed with ECN carrying the *sat* gene deletion (Δ *sat* ECN) had significantly less neurodegeneration compared to those fed with WT ECN ( Figure 1A ), suggesting that ECN Sat contributes to αS-induced DA neuronal loss. To verify the role of SAT in neurodegeneration, we added WT ECN supernatant to the Δ *sat* ECN plates to feed animals and observed return of DA neurodegeneration to levels seen in WT ECN ( Figure 1B ), which supports the previous findings that Sat is secreted (Toloza et al., 2015) . Using a 3 kDa molecular weight cutoff centrifugal filter to separate components in the WT ECN supernatant, we found that only the > 3 kDa fraction restored neurodegeneration with Δ *sat* ECN ( Figure 1B ). Additionally, heating the supernatant at 95°C or digesting with Proteinase K ablated the neurodegenerative activity of WT ECN supernatant ( Figure 1B ), supporting that the Sat protein is secreted and responsible. Notably, ECN Sat, but not Δ *sat* ECN, can also induce spontaneous DA loss in TG2435 animals ( Figure 1C ), which expresses only GFP in dopaminergic (DA) neurons under the control of the *dat-1* promoter, indicating that Sat alone can cause damage to healthy DA neurons. We also examined whether ECN Sat affects other neurodegenerative disease models. In a *C. elegans* Alzheimer's disease (AD) model CL2006 , which expresses human amyloid-β in the muscle tissues that causes paralysis of animals (Wu et al., 2006), Δ *sat* ECN-fed animals showed significantly less paralysis than WT ECN-fed animals ( Figure 1D ), suggesting that Sat plays a role in exacerbating amyloid-β-induced muscle dysfunction. Similarly, in a tauopathy model BR5707 (Fatouros et al., 2012) , in which human pro-aggregating tau fragment, F3ΔK280, and the full-length mutant tau protein (V337M) are co-expressed in all neurons and the GABAergic motor neurons are labeled by GFP ( *rab-3p::F3ΔK280* ; *aex-3p::h4R1NTauV337M* ; *unc-25 p::GFP* ), we found that Δ *sat* ECN-fed animals exhibited less ventral cord axonal breaks than WT ECN- and OP50-fed animals ( Figure 1E ), indicating that Sat similarly exacerbates Tau-induced neurodegeneration. Overall, we find that ECN Sat consistently enhances neurodegenerative phenotypes in several models we tested, with the only exception being the Huntington's disease model AM101 (Gidalevitz et al., 2006) ( Figure 1F ). Although preliminary in nature, these data suggest that ECN Sat (and potentially all *E. coli* Sat isoforms) may contribute to neurodegeneration. Although these findings, as well as a specific mechanism for Sat-induced neurodegeneration, remain to be verified in mammalian models and human cell lines, we find that Sat is a potential bacterial factor contributing to neurodegenerative disease. Given that ECN products like Mutaflor® are commercialized and used as vectors to design targeted therapeutics against human disease, it may be important to create future ECN-based products with the *sat* gene deleted to minimize the long-term risks of promoting neurodegenerative diseases in humans.

## Methods

***Bacterial strains and supernatant collection.*** *E. coli* strains OP50 and Nissle 1917 (ECN) were used in this study. ECN without *sat* (ECOLIN_16105) ( *i.e.,* Δ *sat* ECN) was kindly provided by S.H. Hong (Fang et al *.,* 2018) and verified using ECN Sat-specific primers compared to WT ECN. *E. coli* strains were cultured using LB broth and seeded on nematode growth medium (NGM) Petri dish plates.

***C. elegans strains and culture conditions.*** *C. elegans* strains were raised at 20℃ on NGM plates seeded with *E. coli* strains OP50 or Nissle (WT or Δ *sat* ) as the food source using standard methods (Brenner, 1974) . The following *C. elegans* strains were used in this study: UA44 , *baIn11 [ dat-1 p::gfp* + *dat-1 p::α-synuclein]* (Kautu et al., 2013) ; TG2435 , *vtIs1* [ *dat-1 p::gfp* + *rol-6 ( su1006 )* ]; CL2006 , *dvIs2* [ *unc-54 p::Aβ1-42* + *rol-6 ( su1006 )* ] (Wu et al *.,* 2006) *;* BR5707 , *byIs161 [rab-3p::F3ΔK280 + myo-2p::mCherry]; bkIs10 [aex-3p::h4R1NTauV337M + myo-2p::gfp]; juIs73 [ unc-25 p::gfp]* (Fatouros et al *.,* 2012); and AM101 , *rmIs110 (F25B3p::Q40::yfp)* (Gidalevitz et al *.,* 2006).

***Quantification of dopaminergic neurodegeneration and supernatant fractionation and treatments.*** Synchronized larval stage 1 (L1) UA44 or TG2435 animals (via bleaching of adult animals and arresting development of larvae in the M9 buffer) were grown on NGM plates seeded with OP50 or ECN strains and analyzed at adult day 2 or day 7 stage. To quantify dopaminergic (DA) neuronal death, the percentage of animals missing at least one of the six DA neurons in the head was determined via fluorescent microscopy. For supernatant treatments, larval stage 4 (L4) animals fed with Δ *sat* ECN were treated with supernatants from overnight WT ECN cultures in LB broth, which were collected by centrifugation at 4000 x g for 10 minutes at room temperature and then filter-sterilized (0.22 µm). Thereafter, 500 µL supernatant was loaded onto 3 kDa molecular weight cutoff centrifugal filter columns (Amicon® 0291), centrifuged per manufacturer instructions, and each fraction ( *i.e.,* < 3 kDa and > 3 kDa) was adjusted to the original volume with LB broth. Heat-inactivated supernatant was prepared by heating WT ECN supernatant at 95°C for ten minutes and incubated at 20°C for another 10 minutes prior to adding to L4 animals. Proteinase K experiments were performed by incubating WT ECN supernatant with Proteinase K (250 µg/mL) for 60 minutes at 20°C. Three biological experiments were independently performed (n = 50 per group).

***Paralysis assays in CL2006 animals.*** In CL2006 animals, which constitutively express human amyloid-β under the control of the *unc-54* gene promoter, exhibit a progressive paralysis phenotype (Wu et al *.,* 2006). Synchronized L1 CL2006 animals were grown on NGM plates seeded with *E. coli* strain of interest and analyzed for paralysis at adult day 1 stage. Animals were verified via gentle prodding for the paralysis phenotype, and those that moved following prodding were not considered paralyzed. Three biological experiments were independently performed (n = 50 per group).

***Quantification of axonal breaks in BR5707 animals.*** In the BR5707 strain, GFP expression under the control of the *unc-25* promoter enables microscopic visualization of ventral and dorsal nerve cords, and tau aggregation induces axonal breaks (Fatouros et al *.,* 2012). Synchronized L1 BR5707 animals were grown on NGM plates seeded with *E. coli* of interest, and total axonal breaks were scored at adult day 7 via fluorescent microscopy. Three biological experiments were independently performed (n = 50 per group).

***Quantification of Q40 aggregates in AM101 animals.*** The AM101 strain models Huntington's disease via transgenic expression of polyglutamine (Q40) tagged with YFP in all neurons (Gidalevitz et al *.,* 2006). Synchronized L1 AM101 animals were grown on NGM plates seeded with *E. coli* of interest, and total Q40::YFP levels in the head region were quantified at adult day 7 and 13 stages via fluorescent microscopy and ImageJ analysis. Three biological experiments were independently performed (n = 15 per group).

***Statistical analysis.*** All statistical analyses were performed using GraphPad PRISM version 10.3.1. For all experiments, one-way ANOVAs followed by Tukey's multiple comparisons tests were used to compare differences between bacterial diet, age,and WT ECN treatments.

## Reagents

**Table:** 

**Bacterial Strains**	**Genotype**	**Source**
*Escherichia coli* OP50	WT	CGC
*Escherichia coli* Nissle 1917	WT	M. Mellata (Iowa State University)
*Escherichia coli* Nissle 1917 (Δ *sat* )	*sat* gene deletion	S.H. Hong (Illinois Institute of Technology
***C. elegans* Strains **	**Genotype**	**Source**
UA44	*baIn11* [ *dat-1 p::gfp* + *dat-1 p::α-synuclein* ]	G. Caldwell (University of Alabama)
TG2435	*vtIs1* [ *dat-1 p::gfp* + *rol-6 ( su1006 )* ]	CGC
CL2006	*dvIs2 [ unc-54 p::Aβ1-42)+ rol-6 ( su1006 )] *	CGC
BR5707	*byIs161 [rab-3p::F3ΔK280 + myo-2p::mCherry]; bkIs10 [aex-3p::h4R1NTauV337M + myo-2p::GFP]; juIs73 [ unc-25 p::GFP] *	R. Baumeister (University of Freiburg)
AM101	*rmIs110* [ *F25B3p::Q40::yfp* ]	CGC
